# Monitoring Road Accidents and Injuries Using Variance Chart under Resampling and Having Indeterminacy

**DOI:** 10.3390/ijerph18105247

**Published:** 2021-05-14

**Authors:** Muhammad Aslam, Mohammed Albassam

**Affiliations:** Department of Statistics, Faculty of Science, King Abdulaziz University, Jeddah 21551, Saudi Arabia; malbassam@kau.edu.sa

**Keywords:** road injuries, road accident, monitoring, shift, variance

## Abstract

The current manuscript proposes a $S_{N}^{2}-NEWMA$ control chart for monitoring road accidents and injuries using repetitive sampling. The proposed chart helps in identifying the shifts in accidents and injuries more quickly than existing charts. The application of the proposed chart will help in reducing and identifying the reasons for road accidents and road injuries efficiently.

## 1. Introduction

Control charts are designed to indicate a shift in the process and help industrialists, services companies, and the policy-makers department brings back the process to its normal state. Control charts have the ability to give prior information on when, on average, the process is going to be out-of-control. Therefore, control charts are wonderful tools to minimize the non-conforming items and increase the profit of industry and service companies. Control charts guide policy-makers in identifying the source of variations that cause the shift in the process from the center. References [1,2] discussed the applications of control charts.

Control charts have been broadly applied in monitoring road accidents, road injuries, and road crashes. Control charts lead highway experts in designing roads to minimize road accidents and injuries. In addition, these are helpful in identifying the factors that cause an increase in road accidents and injuries. The proper monitoring of roads with the help of control charts may significantly reduce road accidents and crashes. The application of control charts in monitoring children’s road injuries was discussed by [3]. The various aspects of road accidents with the help of a control chart were discussed by [4]. References [5,6,7,8] presented the applications of control charts in monitoring road accidents. [1] introduced an exponentially weighted moving average (EWMA) for monitoring road accidents. A good statistical analysis of road accident data was discussed by [9,10]. Reference [11] presented the control charts for monitoring hazardous road accidents. Reference [12] presented the control charts using the Saudi traffic accidents data. [13,14] presented the statistical analysis by using motorcyclist injuries data and road accident data. References [15,16] presented excellent work in monitoring road accidents.

The neutrosophic logic, which is an extension of the fuzzy logic, is applied when indeterminacy is presented in the data [17]. According to [18], neutrosophic logic is more efficient than fuzzy logic and interval-based analysis. Reference [17] argued that neutrosophic statistics are more efficient than classical statistics in terms of the measurement of indeterminacy. Reference [19] proposed the neutrosophic EWMA (NEWMA) control chart for monitoring road accidents. Some other applications of neutrosophic statistics can be seen in [20,21,22]. Reference [23] worked on fuzzy-based non-parametric tests. Reference [24] proposed the median test using fuzzy logic. Reference [25] proposed the life-test using the fuzzy approach. Reference [26] proposed the idea of correlation using the fuzzy sets theory. Reference [27] proposed the signed-rank test for the interval data, and [28] presented the correlation analysis using the Pythagorean fuzzy approach. Reference [29] contributed excellent work in making control charts using functional data. Reference [30] studied the effects of indeterminacy on the performance of control charts.

Shewhart variance control charts are applied to monitor the variation in data. The EWMA variance control charts enhance the power of the Shewhart variance control charts, see [31]. References [32,19] introduced control charts under neutrosophic statistics. Reference [33] proposed a $S_{N}^{2}-NEWMA$ chart using a single sampling scheme. Repetitive sampling is the extension of single sampling and is applied when no decision is made on the basis of single sample information. In repetitive sampling, the process of selecting a sample is repeated when no decision is made on the first sample see [34]. To the best of our knowledge, there is still a gap in the design of variance NEWMA charts, $S_{N}^{2}-NEWMA$ being the control chart using repetitive sampling under neutrosophic statistics. In this paper, a $S_{N}^{2}-NEWMA$ control chart using repetitive sampling under neutrosophic statistics will be introduced and applied in monitoring road accidents and road injuries. It is expected that the proposed chart will be more efficient than the existing charts and better help indicate the shift in road accidents and road injuries compared to the existing charts.

## 2. The Proposed $S_{N}^{2}-NEWMA$ Chart

Let $X_{iN}\epsilon \left[X_{L},X_{U}\right],\;i=$1, 2, 3,…,$\;n_{N}$ be a neutrosophic random sample from the neutrosophic normal distribution with a neutrosophic mean of $\mu_{N}\epsilon \left[\mu_{L},\mu_{U}\right]$ and a neutrosophic variance of $\sigma_{N}^{2}\epsilon \left[\sigma_{L}^{2},\sigma_{U}^{2}\right]$, where $n_{N}\epsilon \;\left[n_{L},n_{U}\right]$ is a neutrosophic sample size. Suppose that $\;{\bar{X}}_{N}\;\epsilon \;\left[{\bar{X}}_{L},{\bar{X}}_{U}\right]$ denotes the neutrosophic sample mean and $S_{N}^{2}\;\epsilon \;\left[S_{L}^{2},S_{U}^{2}\right]$ presents the neutrosophic sample variance. Reference [33] proposed the following NEWMA statistic as a generalization of the EWMA statistic proposed by [35,36].

(1) $$Z_{k}{}_{N}=\left(1-\lambda_{N}\right)Z_{k-1,}{}_{N}+\lambda_{N}T_{k}{}_{N};\;Z_{k}{}_{N}\epsilon \;\left[Z_{k}{}_{L},\;Z_{k}{}_{U}\right],\;\lambda_{N}\epsilon \;\left[\lambda_{L},\;\lambda_{U}\right]$$

Note here that $EWMA_{N}=Z_{k}{}_{N}=NEWMA$ and $\lambda_{N}\epsilon \left[\lambda_{L},\lambda_{U}\right]$ are a neutrosophic smoothing constant, selected on the basis of personal experience, [37]. Industrial engineers are always uncertain on the selection of a suitable value for $\lambda_{N}\epsilon \left[\lambda_{L},\lambda_{U}\right]$. Let $I_{N}$ denote the indeterminacy or uncertainty parameter. The neutrosophic form of $\lambda_{N}\epsilon \left[\lambda_{L},\lambda_{U}\right]$ can be expressed as follows

(2) $$\lambda_{N}=\lambda_{L}+\lambda_{U}I_{\lambda N};\;I_{\lambda N}\;\epsilon \;\left[I_{\lambda N},\;I_{\lambda N}\right]$$

Note here that $\lambda_{L}$ denotes the values under classical statistics and is also known as the determined part of the neutrosophic form and $\lambda_{U}I_{\lambda N}$ denotes the indeterminate part of the neutrosophic form. Note here that the neutrosophic form reduces to a smoothing constant under classical statistics when no uncertainty is found in the selection of the smoothing constant.

The values of $T_{k}{}_{N}\epsilon \;\left[T_{k}{}_{L},\;T_{k}{}_{U}\right]$ in Equation (1) can be obtained as follows

(3) $$T_{k}{}_{N}=a_{N}+b_{N}.\ln\left(S_{k}^{2}{}_{N}+c_{N}\right);\;a_{N}\epsilon \;\left[a_{L},a_{U}\right],\;b_{N}\epsilon \;\left[b_{L},b_{U}\right],\;c_{N}\epsilon \;\left[c_{L},c_{U}\right]>\left[0,0\right]$$

Reference [38] showed that $T_{k}{}_{N}\epsilon \;\left[T_{k}{}_{L},T_{k}{}_{U}\right]$ is closer to a neutrosophic normal distribution than $S_{N}^{2}\;\epsilon \;\left[S_{L}^{2},\;S_{U}^{2}\right]$. Reference [39] state: “the main expectation of this approach is that if $a_{N}\epsilon \;\left[a_{L},\;a_{U}\right]$, $b_{N}\epsilon \;\left[b_{L},\;b_{U}\right]$ and $c_{N}\epsilon \;\left[c_{L},\;c_{U}\right]$ are judiciously selected, then this transformation may result in approximate normality to $T_{k}{}_{N}\epsilon \;\left[T_{k}{}_{L},\;T_{k}{}_{U}\right]$”. The neutrosophic control limits (NCLs) under repetitive sampling with starting values of $Z_{0}{}_{N}\;$= 0 are given by:

(4) $$LCL_{1N}=E\left(T_{k}{}_{N}\right)-k_{1N}\sqrt{\frac{\lambda_{N}}{2-\lambda_{N}}}\sigma \left(T_{k}{}_{N}\right);\;LCL_{1N}\epsilon \;\left[LCL_{1L},LCL_{1U}\right]$$

(5) $$UCL_{1N}=E\left(T_{k}{}_{N}\right)+k_{1N}\sqrt{\frac{\lambda_{N}}{2-\lambda_{N}}}\sigma \left(T_{k}{}_{N}\right);\;UCL_{1N}\epsilon \;\left[UCL_{1L},UCL_{1U}\right]$$

(6) $$LCL_{2N}=E\left(T_{k}{}_{N}\right)-k_{2N}\sqrt{\frac{\lambda_{N}}{2-\lambda_{N}}}\sigma \left(T_{k}{}_{N}\right);\;LCL_{2N}\epsilon \;\left[LCL_{2L},LCL_{2U}\right]$$

(7) $$UCL_{2N}=E\left(T_{k}{}_{N}\right)+k_{2N}\sqrt{\frac{\lambda_{N}}{2-\lambda_{N}}}\sigma \left(T_{k}{}_{N}\right);\;UCL_{2N}\epsilon \;\left[UCL_{2L},UCL_{2U}\right]$$

Note that $\;k_{1N}\;\epsilon \;\left[k_{1L},\;k_{1U}\right]$ and $\;k_{2N\;}\epsilon \;\left[k_{2L},\;k_{2U}\right]$ present a neutrosophic control limit coefficient associated with NCLs.

The NCLs given in Equations (1)–(4) are approximate but widely applied due to simplicity, see [39]. The exact NCLs for $S_{N}^{2}-NEWMA$ under repetitive sampling are given as:

(8) $$LCL_{1N}=E\left(T_{k}{}_{N}\right)-k_{1N}\sqrt{\frac{\lambda_{N}\left\{1-{\left(1-\lambda_{N}\right)}^{2k}\right\}}{2-\lambda_{N}}}\sigma \left(T_{k}{}_{N}\right);\;LCL_{N}\epsilon \;\left[LCL_{L},\;LCL_{U}\right]$$

(9) $$UCL_{1N}=E\left(T_{k}{}_{N}\right)+k_{1N}\sqrt{\frac{\lambda_{N}\left\{1-{\left(1-\lambda_{N}\right)}^{2k}\right\}}{2-\lambda_{N}}}\sigma \left(T_{k}{}_{N}\right);\;UCL_{N}\epsilon \;\left[UCL_{L},\;UCL_{U}\right]$$

(10) $$LCL_{2N}=E\left(T_{k}{}_{N}\right)-k_{2N}\sqrt{\frac{\lambda_{N}\left\{1-{\left(1-\lambda_{N}\right)}^{2k}\right\}}{2-\lambda_{N}}}\sigma \left(T_{k}{}_{N}\right);\;LCL_{N}\epsilon \;\left[LCL_{L},\;LCL_{U}\right]$$

(11) $$UCL_{2N}=E\left(T_{k}{}_{N}\right)+k_{2N}\sqrt{\frac{\lambda_{N}\left\{1-{\left(1-\lambda_{N}\right)}^{2k}\right\}}{2-\lambda_{N}}}\sigma \left(T_{k}{}_{N}\right);\;UCL_{N}\epsilon \;\left[UCL_{L},\;UCL_{U}\right]$$

By following [39], the approximate control limits are considered in this paper.

## 3. The Proposed Control Chart

As mentioned in [39], the transformation $T_{k}{}_{N}=\ln S_{N}^{2}$ makes the limits that are not symmetrical in a traditional $S^{2}$ control chart symmetrical. The proposed $S_{N}^{2}-NEWMA$ will be operated as follows:

**Step-1:** Compute statistic $Z_{k}{}_{N}\;\epsilon \;\left[Z_{k}{}_{L},\;Z_{k}{}_{U}\right]$ for $n_{N\;}\epsilon \left[n_{L},\;n_{U}\right]$ sample size when indeterminacy parameter $I_{N}$ is specified.

**Step-2:** If $Z_{k}{}_{N\;}\epsilon \;\left[Z_{k}{}_{L},Z_{k}{}_{U}\right]\geq UCL_{2U}$ or $Z_{k}{}_{N}\epsilon \;\left[Z_{k}{}_{L},\;Z_{k}{}_{U}\right]\leq LCL_{2U}$, the process is said to be out-of-control. The process is said to be in-control if $LCL_{1U}\leq Z_{k}{}_{N}\leq UCL_{1U}$, otherwise repeat step 1.

The proposed control chart has four control limits. The proposed control chart reduces to the control chart proposed by [33] when no repetition is needed. The probability of being in-control for the proposed control chart is:

(12) $$P_{out,N}^{0}=\frac{P_{out,1N}^{0}}{1-P_{rep,N}^{0}}$$

where $P_{rep,N}^{0}$ is the probability of repetition and $P_{out,1N}^{0}$ is the probability of being in-control for the single sampling, given by:

(13) $$P_{out,1N}^{0}=P\left(LCL_{1U}\leq Z_{k}{}_{N}\leq UCL_{1U}/S_{N0}^{2}\right);\;S_{N0}^{2}\epsilon \left[S_{L0}^{2},S_{U0}^{2}\right]$$

The probability of being in-control for the shifted process is given by

(14) $$P_{out,N}^{1}=\frac{P_{out,1N}^{1}}{1-P_{rep,N}^{1}}$$

where $P_{rep,N}^{1}$ is the probability of repetition and $P_{out,1N}^{1}$ is the probability of in-control for the single sampling, given by:

(15) $$P_{out,1N}^{1}=P\left(LCL_{1U}\leq Z_{k}{}_{N}\leq UCL_{1U}/S_{N1}^{2}\right);\;S_{N1}^{2}\epsilon \left[S_{L1}^{2},S_{U1}^{2}\right]$$

The neutrosophic average run length (NARL) for the in-control and shifted process are given by

(16) $$ARL_{0N}=\frac{1}{1-P\left(LCL_{N}\leq Z_{k}{}_{N}\leq UCL_{N}/S_{N0}^{2}\right)};\;ARL_{0N}\epsilon \left[ARL_{0L},ARL_{0U}\right]$$

(17) $$ARL_{1N}=\frac{1}{1-P\left(LCL_{N}\leq Z_{k}{}_{N}\leq UCL_{N}/S_{N1}^{2}\right)};\;ARL_{1N}\epsilon \left[ARL_{1L},ARL_{1U}\right]$$

The following is the neutrosophic Monte Carlo (NMS) used to find the values of $\;k_{1N}\epsilon \;\left[k_{1L},k_{1U}\right]$,$\;k_{2N}\epsilon \;\left[k_{2L},\;k_{2U}\right]$ and $ARL_{1N}\epsilon \left[ARL_{1L},\;ARL_{1U}\right]$, when $ARL_{0N}\epsilon \left[ARL_{0L},\;ARL_{0U}\right]$ is fixed.

1. Fix the sample size $n_{N}\epsilon \left[n_{L},\;n_{U}\right]$ and generate 10,000 random samples of size $n_{N}\epsilon \left[n_{L},\;n_{U}\right]$ and select the values of $a_{N}\epsilon \;\left[a_{L},\;a_{U}\right]$, $b_{N}\epsilon \;\left[b_{L},b_{U}\right]$ and $c_{N}\epsilon \;\left[c_{L},\;c_{U}\right]$ from [33]. Compute the values of the statistic $Z_{k}{}_{N}\epsilon \;\left[Z_{k}{}_{L},\;Z_{k}{}_{U}\right]$ for the specified indeterminacy parameter $I_{N}$ and plot these values of NCLs.
2. Note the first out-of-control values for the 10,000 random samples and compute $ARL_{0N}\epsilon \left[ARL_{0L},ARL_{0U}\right]$ and neutrosophic standard division (NSD) and select the values of $\;k_{1N}\epsilon \;\left[k_{1L},k_{1U}\right]$ and $k_{2N}\epsilon \;\left[k_{2L},k_{2U}\right]$ for which $ARL_{0N}\epsilon \left[ARL_{0L},ARL_{0U}\right]$ is very close to the specified values of $ARL_{0N}\epsilon \left[ARL_{0L},ARL_{0U}\right]$.
3. Using the selected values of $\;k_{1N}\epsilon \;\left[k_{1L},k_{1U}\right]$ and $k_{2N}\epsilon \;\left[k_{2L},k_{2U}\right]$, compute $Z_{k}{}_{N}\epsilon \;\left[Z_{k}{}_{L},Z_{k}{}_{U}\right]$ for the data generated at various values of shift c. Compute the values of $ARL_{1N}\epsilon \left[ARL_{1L},ARL_{1U}\right]$ and NSD for various values of c.

Using the above algorithm, the values of $ARL_{1N}\epsilon \left[ARL_{1L},ARL_{1U}\right]$ and NSD for various values of c, $ARL_{0N}\epsilon \left[ARL_{0L},ARL_{0U}\right]$, $n_{N}\epsilon \;\left[n_{L},n_{U}\right]$ and $I_{N}$ are placed in Table 1, Table 2, Table 3, Table 4, Table 5, Table 6. Table 1 is presented for $n_{N}\epsilon$ [3,5] and $\lambda_{N}=0.08+0.12I_{\lambda N};I_{\lambda N}\epsilon \left[0,\;0.3\right]$. Table 2 is given for $n_{N}\epsilon$ [3,5] and $\lambda_{N}=0.18+0.22I_{\lambda N};I_{\lambda N}\epsilon \left[0,\;0.18\right]$. Table 3 is given for $n_{N}\epsilon$ [3,5] and $\lambda_{N}=0.28+0.32I_{\lambda N};I_{\lambda N}\epsilon \left[0,\;0.13\right]$. Table 4 is presented for $n_{N}\epsilon$ [8,10] and $\lambda_{N}=0.08+0.12I_{N};I_{N}\epsilon \left[0,\;0.3\right]$. Table 5 is given for $n_{N}\epsilon$ [8,10] and $\lambda_{N}=0.18+0.22I_{\lambda N};I_{\lambda N}\epsilon \left[0,0.18\right]$. Finally, Table 6 is given for $n_{N}\epsilon$ [8,10] and $\lambda_{N}=0.28+0.32I_{\lambda N};I_{\lambda N}\epsilon \left[0,\;0.13\right]$. The R codes to make the Tables are given in Appendix A.

From Table 1, Table 2, Table 3, Table 4, Table 5 and Table 6, the following trends can be observed.

1. For other same parameters, the values of $ARL_{1N}\epsilon \left[ARL_{1L},ARL_{1U}\right]$ and NSD increase as the values of $I_{\lambda U}$ decrease.
2. For the other same parameters, $ARL_{1N}\epsilon \left[ARL_{1L},ARL_{1U}\right]$ and NSD decrease as the values of $n_{N}\epsilon \;\left[n_{L},n_{U}\right]$ increase.
3. The values of $ARL_{1N}\epsilon \left[ARL_{1L},ARL_{1U}\right]$ and NSD decrease as the value of the parameter c increases from 1.00 to 4.0.
4. For the same value of c, the values of $ARL_{1N}\epsilon \left[ARL_{1L},ARL_{1U}\right]$ and NSD increase as the value of $ARL_{0N}$ increases.

## 4. Comparative Study

In this section, the advantage of the proposed control chart is discussed in terms of NARLs and NSD. The proposed chart is compared to two existing control charts proposed by [39] under classical statistics and [33] under neutrosophic statistics. The same values of all parameters are used to compare the performance of the proposed control. The values of NARLs and NSD of the three control charts when $n_{N}\epsilon$ [3,5] and $n_{N}\epsilon$ [8,10] are shown in Table 7.

.

From Table 7, it is clear that the proposed control chart provides smaller values of NARLs compared to [39,33] control charts. For example, when c = 1.05 and $n_{N}\epsilon$ (8, 10), the values of ARL and SD from [39] control chart are 109 and 106, respectively. The values of NARL and NSD from [33] control chart are from 107 to 109 and 102 to 104, respectively. The values of NARL and NSD for the proposed control are from 92 to 100 and 91 to 102, respectively. From this study, it can be seen that the control chart proposed by [39] detects the shift in the process at the 106th sample. The control chart proposed by [33] detects the shift from the 92nd sample and 104th sample. It is quite clear that the proposed chart detects the shift in the process quicker than the existing control charts. From this study, it can be concluded that the use of the proposed control chart may reduce road injuries and road accidents. The proposed chart has the ability to point out the cause of variations for road injuries and road accidents as early as possible.

### Road Accidents and Injuries Monitoring Using Simulated Data

**Figure 1:**
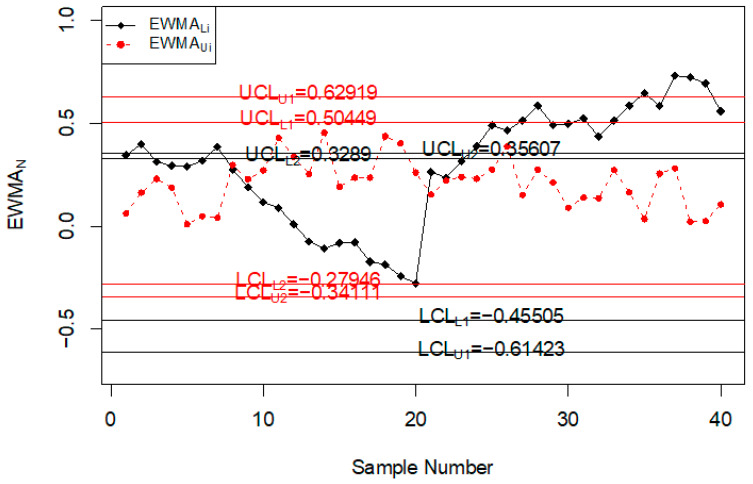
Proposed control chart for simulated data set.

**Figure 2:**
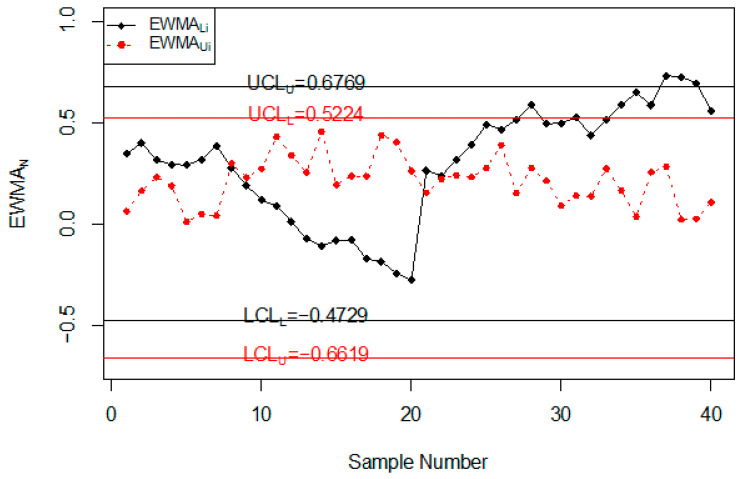
The control chart by [33] for simulated data set.

**Figure 3:**
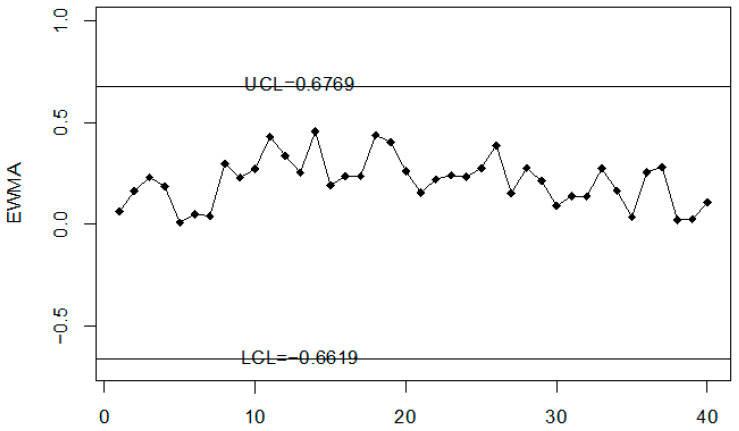
The control chart by [39] for simulated data set.

In this section, the performance of the proposed chart for monitoring road accidents and injuries is discussed using the simulated data. The simulated data is generated from the neutrosophic normal distribution. It is assumed that the process is in-control at neutrosophic variance $S_{N0}^{2}\in \left[1,\;1\right]$. The first 20 values are generated at $S_{N0}^{2}\in \left[1,\;1\right]$ and the next 20 values are generated from the shifted process when c = 1.25, $n_{N}\in \left[3,\;5\right]$ and $\lambda_{N}\in \left[0.08,\;0.12\right]$. The values of the neutrosophic statistic $Z_{kN}\in \left[Z_{kL},\;Z_{kU}\right]$ are calculated for the proposed chart, [39] chart and [33] chart and are plotted on control charts in Figure 1, Figure 2 and Figure 3. Figure 1 shows the proposed control chart, Figure 2 shows the control chart by [33], and Figure 3 depicts [39] control chart. At the specified parameters, the proposed chart should detect the shift in the process from the 9th sample to the 15th sample. From Figure 1, it is clear that the proposed chart detects the shift from the 9th sample to the 15th sample as expected. In addition, several points are within indeterminacy intervals. The existing chart proposed by [33] detects a shift at the 36th sample. The control chart proposed by [39] does not detect any shift in the process. The simulation study showed that the proposed control chart detected a shift in road accidents and injuries earlier than the existing charts. The use of the proposed control chart will be helpful in minimizing the number of road accidents and injuries.

## 5. Road Accidents and Injuries Monitoring Using Real Data

In this section, the application of the proposed control chart is given with the help of two real examples. The real data of injuries and accidents in Saudi Arabia were collected from https://data.gov.sa/Data/en/dataset/1439/resource/e6a973aa-32a8-4fa2-964c-78bcf0e8bf58 (accessed on 16 October 2020). The monitoring of road injuries using the proposed control chart and existing charts by [33,39] is discussed in example 1. Example 2 shows the control chart for monitoring the number of accidents using the proposed control chart and the existing control charts proposed by [33,39].

### 5.1. Example 1: Monitoring the Injuries

For the real-life application of the proposed chart, the injury data of various age ranges of people in different months of the year are reported in Table 8. The injury of people in various months of the year is a variable of interest here. The data are shown in Table 8. The calculations of the statistic of $T_{kN}\in \left[T_{kL},\;T_{kU}\right],\;Z_{kN}\in \left[Z_{kL},\;Z_{kU}\right]$ when $n_{N}\in \left[5,5\right]$ and $\lambda_{N}\in \left[0.08,\;0.12\right]$ are also shown in Table 8.

**Figure 4:**
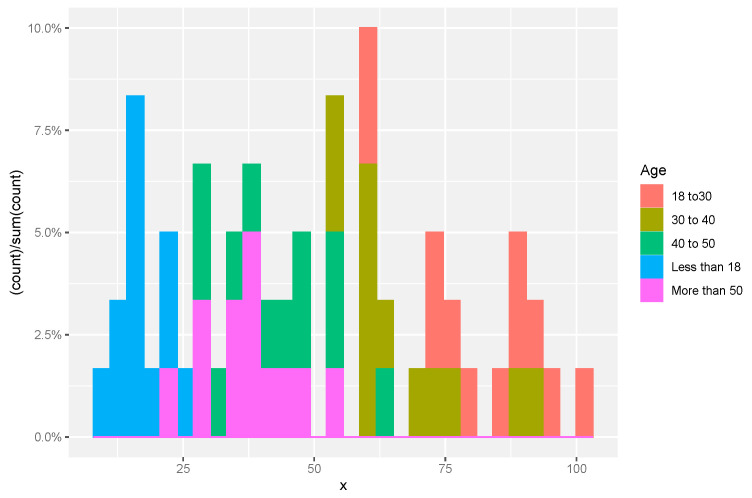
A histogram represents the road accident Injury level with different age group.

**Figure 5:**
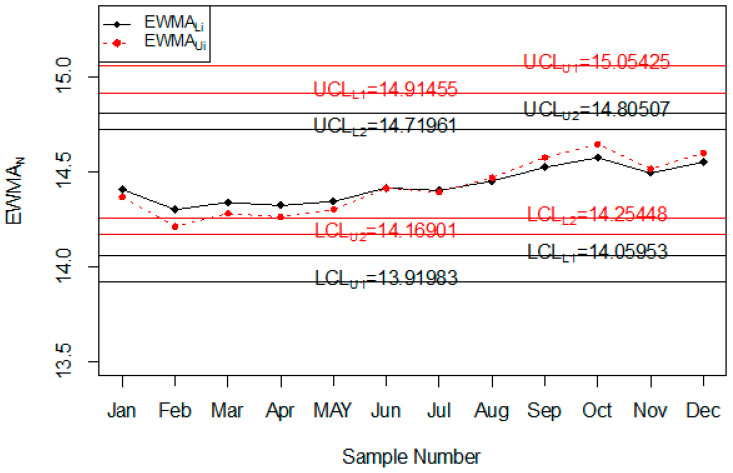
The Proposed control chart for injuries data.

**Figure 6:**
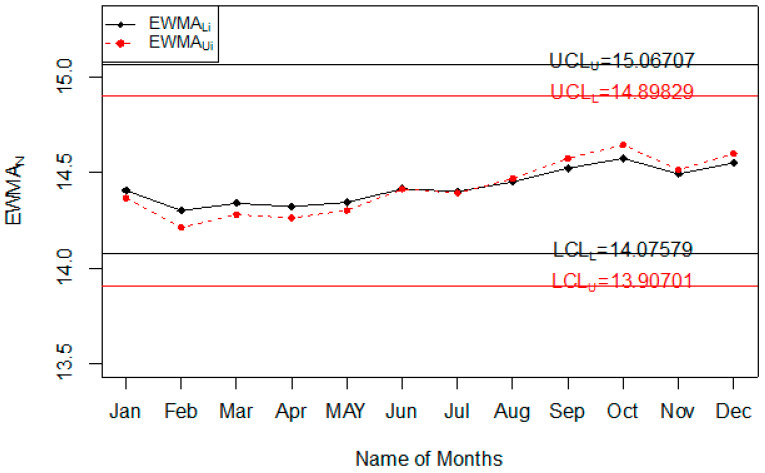
Chart for injuries data.

**Figure 7:**
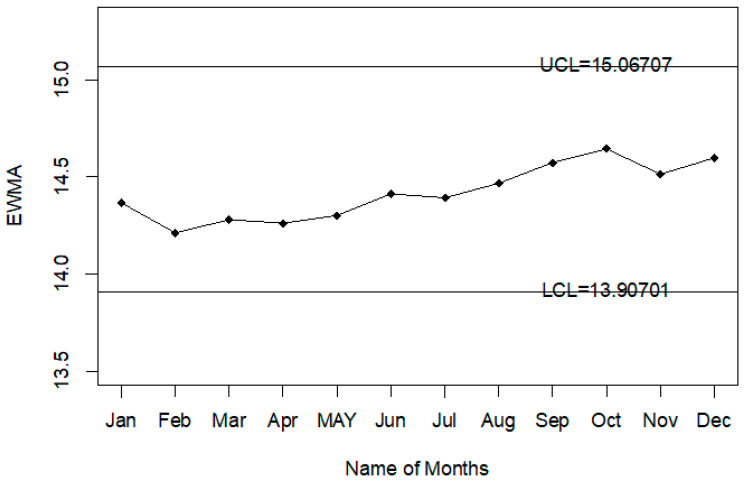
Chart for injuries data.

The Injury level with the different age range in the whole year is shown in Figure 4. So, it can be seen that most people that are injured during road accidents are aged from 18 years to 30 years; less injury is recorded in people whose age is less than 18 years or more than 50 years. The application of the proposed control chart and the two existing charts is also shown using the control chart figures. The monitoring of road injuries using the proposed control chart is shown in Figure 5. The control chart proposed by [33] for the injuries data is shown in Figure 6. Figure 7 presents the control chart proposed by [39]. From Figure 6, it can be noted that some points are in indeterminate intervals and several points are near control limits which are indicating that there may be a shift in the injuries. On the other hand, Figure 6 and Figure 7 show that the number of injuries is within control, and these charts are not indicating any issue in the process. By comparing Figure 5 of the proposed chart with Figure 6 and Figure 7 of the existing control charts, it can be concluded that the proposed chart shows that the decision-makers can expect a shift in road injuries. Therefore, they should be alert and identify the factors for this shift in the process.

### 5.2. Example 2: Monitoring Road Accidents

**Figure 8:**
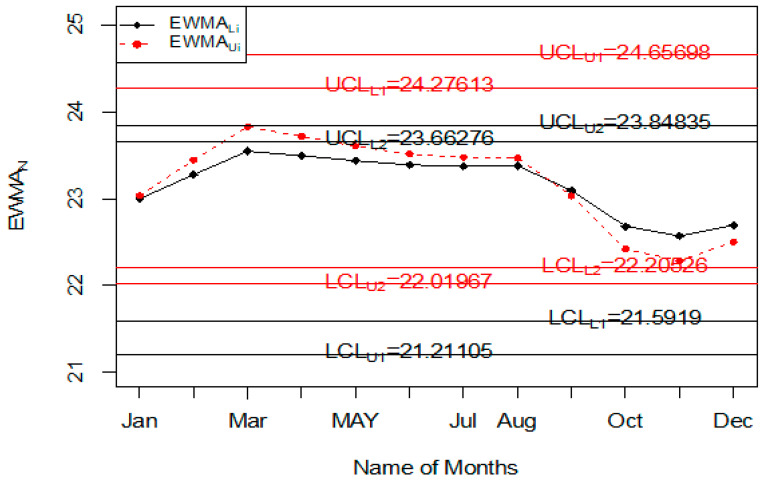
The proposed control chart for accidents data.

**Figure 9:**
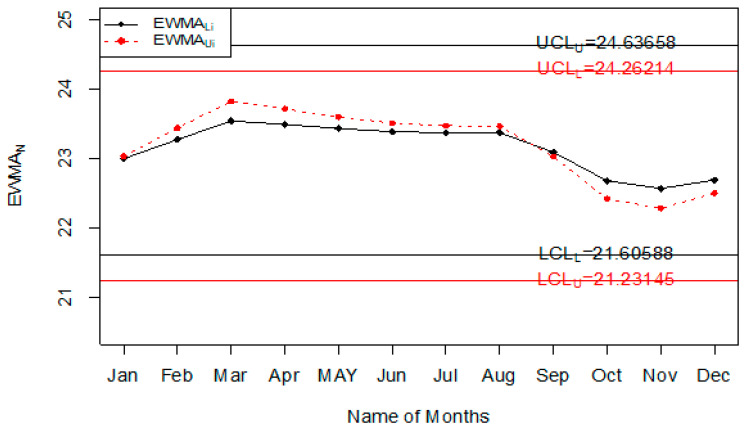
Chart for accidents data [33].

**Figure 10:**
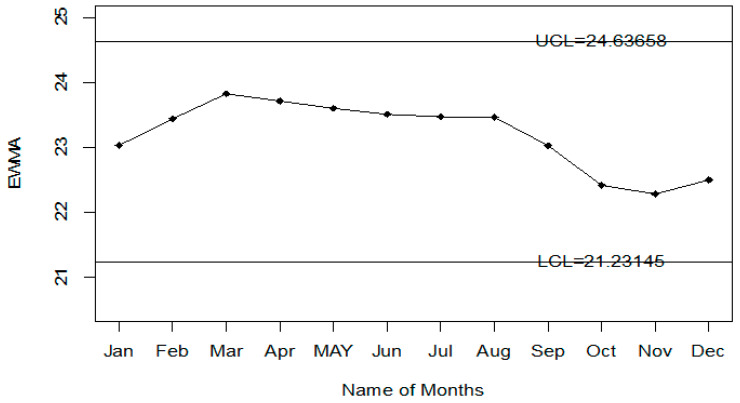
Chart for accidents data set.

For the application of the proposed chart, the road accident data of all days and weeks of the year are used. The purpose of this example is to monitor road accidents on various days of the week. The data of road accidents are shown in Table 9. The values of the statistic of $T_{kN}\in \left[T_{kL},\;T_{kU}\right],\;Z_{kN}\in \left[Z_{kL},\;Z_{kU}\right]$ when $n_{N}\in \left[5,\;5\right]$ and $\lambda_{N}\in \left[0.08,\;0.12\right]$ are also reported in Table 9. The application of the proposed control chart and two existing charts are also shown using the control chart Figures. The monitoring of road accidents using the proposed control chart is shown in Figure 8. The control chart proposed by [33] for road accidents data is shown in Figure 9. Figure 10 presents the control chart for road accidents proposed by [39]. From Figure 8, it can be noted that some points are in indeterminate intervals and several points are near control limits which are indicating that there may be a shift in road accidents. On the other hand, Figure 9 and Figure 10 show that the number of road accidents is within control, and these charts are not indicating any issue in the process. By comparing Figure 8 of the proposed chart with Figure 9 and Figure 10 of the existing control charts, it can be concluded that the proposed chart shows that the decision-makers can expect a shift in road accidents. Therefore, they should be alert and identify the factors that cause the shift in road accidents.

## 6. Concluding Remarks

The $S_{N}^{2}-NEWMA$ control chart for monitoring road accidents and road injuries when the smoothing constant is uncertain was presented in the paper. The operational procedure of the proposed chart was explained. The neutrosophic Monte Carlo simulation for the proposed control chart was introduced and used to present the Tables and control chart Figures. The comparative study showed that the proposed control chart is quite an effective tool in monitoring road accidents and road injuries. The efficiency of the proposed control chart was shown over two control charts in terms of NARL. Based on the analysis, it is recommended to apply the proposed control chart in monitoring highways and motorways to minimize road accidents and injuries. The proposed control chart using a cost model can be studied as future research. The proposed control chart using the rank set sampling scheme is also a fruitful area for future research. The seasonal trend of the series of accidents and injuries can be studied as future research.

## Figures and Tables

**Table 1:** The NARL when $n_{N}\epsilon$ [3,5] and $I_{\lambda U}=0.3$.

c	k_1_ = [2.60,2.807], k_2_ = [1.65,1.622], ARL_0N_ = [300,300]		k_1_ = [2.746,2.875], k_2_ = [1.741,1.612], ARL_0N_ = [370,370]	
	NARL	NSD	NARL	NSD
1.00	[306.67,300.58]	[311.79,294.36]	[370.27,370.12]	[398.78,362.83]
1.05	[131.79,116.61]	[139.41,124.24]	[158.75,135.45]	[168.84,142.24]
1.1	[63.80,44.18]	[70.27,46.39]	[77.63,51.89]	[89.02,52.37]
1.15	[33.24,22.42]	[37.08,22.88]	[36.97,24.85]	[40.83,24.06]
1.2	[21.35,13.50]	[23.31,13.65]	[22.66,12.96]	[25.26,12.71]
1.25	[14.13,8.51]	[15.65,8.30]	[15.05,9.36]	[17.21,8.72]
1.3	[9.43,6.45]	[10.03,5.75]	[10.88,6.06]	[12.18,5.59]
1.4	[5.97,4.01]	[6.17,3.31]	[6.75,4.07]	[6.94,3.47]
1.5	[4.28,2.99]	[4.46,2.40]	[4.46,2.99]	[4.20,2.29]
1.6	[3.23,2.31]	[3.21,1.56]	[3.66,2.36]	[3.20,1.60]
1.7	[2.69,2.07]	[2.35,1.34]	[2.91,2.05]	[2.42,1.28]
1.8	[2.23,1.75]	[1.87,1.02]	[2.56,1.75]	[2.15,1.05]
1.9	[2.02,1.61]	[1.64,0.94]	[2.21,1.63]	[1.82,0.92]
2.0	[1.96,1.49]	[1.56,0.76]	[1.88,1.54]	[1.34,0.81]
2.25	[1.55,1.31]	[0.98,0.61]	[1.67,1.33]	[1.06,0.60]
2.5	[1.46,1.20]	[0.84,0.46]	[1.54,1.22]	[1.03,0.49]
3.0	[1.27,1.10]	[0.58,0.33]	[1.30,1.13]	[0.67,0.38]
4.0	[1.13,1.03]	[0.40,0.19]	[1.15,1.03]	[0.43,0.18]

**Table 2:** The NARL when $n_{N}\epsilon$ [3,5] and $I_{\lambda U}=0.18$.

c	k_1_ = [2.746,2.887], k_2_ = [1.741,1.612], ARL_0N_ = [300,300]		k_1_ = [2.824,2.949], k_2_ = [1.782,1.726], ARL_0N_ = [370,370]	
	NARL	NSD	NARL	NSD
1.00	[302.77,299.08]	[322.28,279.96]	[371.30,370.76]	[380.06,342.35]
1.05	[140.91,126.91]	[149.12,131.00]	[170.67,144.53]	[176.01,148.56]
1.1	[76.46,51.85]	[77.38,51.80]	[89.82,63.61]	[90.73,62.82]
1.15	[46.43,27.69]	[47.98,26.04]	[47.37,29.93]	[50.06,28.22]
1.2	[27.10,17.24]	[28.23,16.96]	[31.76,17.83]	[32.01,17.18]
1.25	[19.05,10.37]	[19.25,10.35]	[20.88,11.00]	[21.02,10.08]
1.3	[13.37,7.00]	[12.30,6.07]	[13.72,8.27]	[14.65,7.51]
1.4	[8.43,4.58]	[8.15,3.84]	[8.95,4.87]	[7.94,4.07]
1.5	[5.83,3.29]	[5.28,2.60]	[5.97,3.37]	[5.49,2.55]
1.6	[4.46,2.53]	[4.00,1.84]	[4.97,2.74]	[4.26,1.82]
1.7	[3.60,2.17]	[3.03,1.40]	[3.78,2.26]	[3.16,1.49]
1.8	[3.22,1.90]	[2.74,1.24]	[3.24,2.03]	[2.52,1.30]
1.9	[2.63,1.76	[2.02,1.08]	[2.79,1.78]	[2.10,1.06]
2.0	[2.49,1.53]	[1.91,0.83]	[2.65,1.65]	[1.91,0.91]
2.25	[2.06,1.33]	[1.36,0.59]	[2.06,1.38]	[1.37,0.64]
2.5	[1.75,1.21]	[1.07,0.50]	[1.76,1.25]	[1.09,0.54]
3.0	[1.39,1.13]	[0.70,0.38]	[1.49,1.11]	[0.86,0.33]
4.0	[1.23,1.04]	[0.52,0.22]	[1.21,1.04]	[0.49,0.21]

**Table 3:** The NARL when $n_{N}\epsilon$ [3,5] and $I_{\lambda U}=0.13$.

c	k_1_ = [2.823,2.888], k_2_ = [1.782,1.687], ARL_0N_ = [300,300]		k_1_ = [2.907,2.958], k_2_ = [1.752,1.617], ARL_0N_ = [370,370]	
	NARL	NSD	NARL	NSD
1.00	[299.03,300.32]	[311.97,281.24]	[369.70,370.73]	[374.55,352.17]
1.05	[142.14,129.44]	[143.09,127.88]	[164.25,135.02]	[165.41,132.88]
1.1	[78.27,54.44]	[75.26,55.67]	[93.17,65.74]	[93.99,65.19]
1.15	[47.16,31.04]	[46.35,29.41]	[52.44,31.17]	[54.35,29.80]
1.2	[31.19,19.61]	[31.68,18.60]	[33.70,19.81]	[33.91,19.41]
1.25	[20.81,12.15]	[20.72,12.08]	[22.70,12.02]	[22.56,11.24]
1.3	[15.35,8.17]	[15.29,7.30]	[16.04,8.86]	[15.96,7.97]
1.4	[8.89,5.09]	[8.02,4.38]	[9.77,5.03]	[9.41,4.04]
1.5	[6.44,3.61]	[5.99,2.94]	[6.66,3.56]	[5.89,2.69]
1.6	[5.02,2.75]	[4.38,2.03]	[5.01,2.60]	[4.47,1.85]
1.7	[3.80,2.26]	[3.19,1.51]	[4.07,2.17]	[3.24,1.45]
1.8	[3.25,2.00]	[2.76,1.31]	[3.35,1.92]	[2.61,1.31]
1.9	[2.91,1.77]	[2.27,1.13]	[2.90,1.67]	[2.27,0.92]
2.0	[2.53,1.59]	[1.92,0.89]	[2.47,1.61]	[1.92,0.89]
2.25	[2.06,1.36]	[1.43,0.63]	[2.04,1.34]	[1.40,0.65]
2.5	[1.76,1.23]	[1.11,0.51]	[1.73,1.24]	[1.10,0.51]
3.0	[1.48,1.13]	[0.87,0.36]	[1.48,1.09]	[0.84,0.32]
4.0	[1.23,1.05]	[0.53,0.23]	[1.25,1.04]	[0.57,0.21]

**Table 4:** The NARL when $n_{N}\epsilon$ [8,10] and $I_{\lambda U}=0.3$.

c	k_1_ = [2.756,2.899], k_2_ = [1.467,1.452], ARL_0N_ = [300,300]		k_1_ = [2.849,2.968], k_2_ = [1.567,1.452], ARL_0N_ = [370,370]	
	NARL	NSD	NARL	NSD
1.00	[301.25,300.24]	[289.01,302.93]	[369.02,371.91]	[355.43,378.85]
1.05	[88.50,86.94]	[84.53,84.26]	[100.77,92.29]	[102.60,91.54]
1.1	[25.72,23.21]	[25.30,22.29]	[27.70,24.97]	[27.70,24.51]
1.15	[11.03,9.93]	[11.08,9.08]	[12.00,10.25]	[11.50,9.08]
1.2	[6.33,5.66]	[5.78,4.73]	[6.86,5.61]	[5.81,4.30]
1.25	[4.28,3.99]	[3.43,3.01]	[4.77,3.90]	[3.94,2.86]
1.3	[3.46,3.06]	[2.61,2.17]	[3.78,2.97]	[2.78,2.09]
1.4	[2.45,2.12]	[1.71,1.24]	[2.49,2.10]	[1.66,1.31]
1.5	[1.90,1.79]	[1.18,1.00]	[2.00,1.70]	[1.23,0.89]
1.6	[1.67,1.49]	[0.98,0.75]	[1.71,1.48]	[0.92,0.72]
1.7	[1.44,1.33]	[0.69,0.57]	[1.51,1.35]	[0.79,0.62]
1.8	[1.32,1.25]	[0.59,0.50]	[1.39,1.26]	[0.65,0.54]
1.9	[1.26,1.17]	[0.54,0.44]	[1.33,1.17]	[0.58,0.42]
2.0	[1.23,1.13]	[0.49,0.35]	[1.20,1.12]	[0.45,0.35]
2.25	[1.08,1.07]	[0.29,0.27]	[1.13,1.07]	[0.37,0.27]
2.5	[1.06,1.03]	[0.24,0.18]	[1.07,1.03]	[0.27,0.20]
3.0	[1.01,1.01]	[0.11,0.11]	[1.02,1.01]	[0.15,0.09]
4.0	[1.00,1.00]	[0.05,0.03]	[1.00,1.00]	[0.07,0.00]

**Table 5:** The NARL when $n_{N}\epsilon$ [8,10] and $I_{\lambda U}=0.18$.

c	k_1_ = [2.929,2.995], k_2_ = [1.637,1.442], ARL_0N_ = [300,300]		k_1_ = [2.989,3.043], k_2_ = [1.687,1.652], ARL_0N_ = [370,370]	
	NARL	NSD	NARL	NSD
1.00	[300.61,301.96]	[289.96,307.92]	[369.57,371.06]	[361.81,374.03]
1.05	[103.60,101.21]	[106.57,99.75]	[124.33,121.34]	[120.92,115.25]
1.1	[38.85,30.55]	[37.33,28.94]	[41.19,36.64]	[40.42,36.47]
1.15	[15.86,12.48]	[14.32,11.60]	[17.62,14.74]	[16.45,13.06]
1.2	[9.16,6.92]	[7.57,5.66]	[9.85,7.74]	[8.71,6.23]
1.25	[5.99,4.51]	[4.85,3.68]	[6.16,4.85]	[4.84,3.82]
1.3	[4.21,3.20]	[3.17,2.49]	[4.61,3.72]	[3.61,2.68]
1.4	[2.93,2.08]	[2.05,1.37]	[3.07,2.57]	[2.13,1.69]
1.5	[2.21,1.76]	[1.33,1.01]	[2.34,1.83]	[1.52,0.98]
1.6	[1.84,1.45]	[1.02,0.69]	[1.86,1.65]	[1.04,0.84]
1.7	[1.57,1.32]	[0.82,0.61]	[1.64,1.39]	[0.88,0.64]
1.8	[1.47,1.20]	[0.73,0.46]	[1.50,1.29]	[0.74,0.53]
1.9	[1.34,1.18]	[0.57,0.44]	[1.37,1.21]	[0.60,0.43]
2.0	[1.26,1.11]	[0.53,0.33]	[1.29,1.15]	[0.55,0.39]
2.25	[1.16,1.04]	[0.38,0.22]	[1.16,1.09]	[0.39,0.30]
2.5	[1.07,1.03]	[0.29,0.17]	[1.06,1.03]	[0.25,0.17]
3.0	[1.03,1.00]	[0.17,0.08]	[1.03,1.00]	[0.18,0.07]
4.0	[1.00,1.00]	[0.07,0.03]	[1.00,1.00]	[0.060.00]

**Table 6:** The NARL when $n_{N}\epsilon$ [8,10] and $I_{\lambda U}=0.13$.

c	k_1_ = [2.977,2.990], k_2_ = [1.653,1.584], ARL_0N_ = [300,300]		k_1_ = [3.037,3.056], k_2_ = [1.852,1.652], ARL_0N_ = [370,370]	
	NARL	NSD	NARL	NSD
1.00	[301.83,300.77]	[301.34,298.27]	[370.70,370.45]	[366.03,361.76]
1.05	[111.33,105.96]	[114.26,105.79]	[142.94,138.04]	[148.14,132.91]
1.1	[46.33,37.46]	[42.97,36.86]	[50.18,42.23]	[49.57,40.51]
1.15	[19.84,15.37]	[17.77,14.55]	[24.12,16.80]	[22.87,16.02]
1.2	[11.10,8.30]	[10.16,7.23]	[12.17,9.60]	[10.83,8.91]
1.25	[6.64,5.35]	[5.46,4.33]	[8.01,5.50]	[7.08,4.49]
1.3	[4.79,3.87]	[3.87,2.94]	[5.54,3.99]	[4.21,2.96]
1.4	[3.00,2.45]	[2.15,1.65]	[3.44,2.58]	[2.40,1.74]
1.5	[2.26,1.80]	[1.49,0.98]	[2.43,1.87]	[1.47,1.07]
1.6	[1.79,1.54]	[1.02,0.83]	[2.05,1.59]	[1.24,0.82]
1.7	[1.57,1.35]	[0.84,0.61]	[1.71,1.42]	[0.91,0.70]
1.8	[1.43,1.22]	[0.74,0.49]	[1.53,1.26]	[0.78,0.53]
1.9	[1.33,1.15]	[0.58,0.40]	[1.37,1.19]	[0.62,0.44]
2.0	[1.21,1.12]	[0.47,0.36]	[1.32,1.12]	[0.58,0.35]
2.25	[1.13,1.05]	[0.38,0.23]	[1.15,1.06]	[0.38,0.24]
2.5	[1.07,1.03]	[0.29,0.17]	[1.09,1.02]	[0.30,0.14]
3.0	[1.02,1.00]	[0.16,0.08]	[1.03,1.00]	[0.19,0.08]
4.0	[1.00,1.00]	[0.07,0.00]	[1.011.00]	[0.11,0.00]

**Table 7:** The NARL values for the proposed chart and existing chart when $I_{\lambda U}=0.3$.

c	[2] Control Chart		[2] Control Chart		[1] Control Chart		[1] Control Chart		Proposed Chart		Proposed Chart	
	3		8		[3,5]		[8,10]		[3,5]		[8,10]	
	ARL	SD	ARL	SD	NARL	NSD	NARL	NSD	NARL	NSD	NARL	NSD
1.00	375.03	366.15	371.14	348.86	[367.88,380.1]	[362.73,361.76]	[375.97,384.4]	[352.21,365.97]	[370.27,370.12]	[398.78,362.83]	[369.02,371.91]	[355.43,378.85]
1.05	175.73	189.55	109.31	106.95	[175.89,155.28]	[184.21,153.18]	[109.14,107.67]	[104.73,102.66]	[158.75,135.45]	[168.84,142.24]	[100.77,92.29]	[102.60,91.54]
1.1	82.72	86.61	38.47	33.1	[81.69,62.06]	[84.91,58.71]	[38.37,35.39]	[32.73,29.6]	[77.63,51.89]	[89.02,52.37]	[27.70,24.97]	[27.70,24.51]
1.15	45.71	46.17	20.48	14.76	[45.63,33.13]	[46.57,29.59]	[20.62,18.75]	[15.19,13.51]	[36.97,24.85]	[40.83,24.06]	[12.00,10.25]	[11.50,9.08]
1.2	29.81	28.95	13.65	8.7	[29.69,21.13]	[28.94,16.94]	[13.68,12.32]	[8.67,7.63]	[22.66,12.96]	[25.26,12.71]	[6.86,5.61]	[5.81,4.30]
1.25	21.01	18.95	10.27	5.87	[20.88,15.26]	[19.43,11.16]	[10.38,9.3]	[5.76,4.91]	[15.05,9.36]	[17.21,8.72]	[4.77,3.90]	[3.94,2.86]
1.3	16.15	13.68	8.38	4.14	[16.2,11.84]	[14.1,7.95]	[8.5,7.5]	[4.3,3.52]	[10.88,6.06]	[12.18,5.59]	[3.78,2.97]	[2.78,2.09]
1.4	11.07	8.56	6.27	2.6	[11.07,8.29]	[8.49,4.83]	[6.3,5.63]	[2.63,2.2]	[6.75,4.07]	[6.94,3.47]	[2.49,2.10]	[1.66,1.31]
1.5	8.52	5.89	5.17	1.88	[8.49,6.56]	[5.92,3.37]	[5.16,4.65]	[1.87,1.58]	[4.46,2.99]	[4.20,2.29]	[2.00,1.70]	[1.23,0.89]
1.6	7.01	4.45	4.51	1.46	[6.96,5.49]	[4.42,2.49]	[4.49,4.05]	[1.45,1.21]	[3.66,2.36]	[3.20,1.60]	[1.71,1.48]	[0.92,0.72]
1.7	6	3.48	4.02	1.18	[5.98,4.78]	[3.51,1.97]	[4.02,3.68]	[1.19,0.99]	[2.91,2.05]	[2.42,1.28]	[1.51,1.35]	[0.79,0.62]
1.8	5.32	2.87	3.72	1.03	[5.37,4.33]	[2.95,1.65]	[3.7,3.38]	[1,0.85]	[2.56,1.75]	[2.15,1.05]	[1.39,1.26]	[0.65,0.54]
1.9	4.87	2.52	3.47	0.88	[4.87,4]	[2.57,1.44]	[3.47,3.15]	[0.89,0.74]	[2.21,1.63]	[1.82,0.92]	[1.33,1.17]	[0.58,0.42]
2.0	4.49	2.19	3.26	0.79	[4.43,3.74]	[2.14,1.26]	[3.28,2.99]	[0.79,0.67]	[1.88,1.54]	[1.34,0.81]	[1.20,1.12]	[0.45,0.35]
2.25	3.8	1.66	2.93	0.67	[3.85,3.27]	[1.7,1.01]	[2.93,2.67]	[0.67,0.59]	[1.67,1.33]	[1.06,0.60]	[1.13,1.07]	[0.37,0.27]
2.5	3.47	1.39	2.7	0.6	[3.44,2.97]	[1.37,0.84]	[2.7,2.47]	[0.6,0.55]	[1.54,1.22]	[1.03,0.49]	[1.07,1.03]	[0.27,0.20]
3.0	2.98	1.08	2.38	0.52	[2.97,2.59]	[1.05,0.67]	[2.38,2.2]	[0.51,0.41]	[1.30,1.13]	[0.67,0.38]	[1.02,1.01]	[0.15,0.09]
4.0	2.53	0.76	2.11	0.32	[2.54,2.27]	[0.75,0.48]	[2.11,2.04]	[0.32,0.19]	[1.15,1.03]	[0.43,0.18]	[1.00,1.00]	[0.07,0.00]

**Table 8:** Real data related to Injury Data of Jeddah.

	Age					$S_{N}^{2}$	$T_{k}{}_{N}$	NEWMA
Months	Less than 18	18 to 30	30 to 40	40 to 50	More than 50			λ = [0.08, 0.12]
January	14	59	62	49	27	436.7	13.482	14.4066, 14.3664
February	11	61	54	39	41	368.2	13.079	14.3004, 14.2119
March	21	92	71	41	48	756.3	14.779	14.3387, 14.2799
April	16	79	61	36	36	575.3	14.1329	14.3223, 14.2623
May	12	74	61	29	23	697.7	14.5886	14.3435, 14.3015
June	18	86	75	33	29	914.7	15.2285	14.4143, 14.4128
July	15	76	61	29	38	603.7	14.2468	14.40091, 14.3928
August	22	89	88	55	44	837.3	15.0196	14.4504, 14.4680
September	25	103	92	62	55	963.3	15.3509	14.5225, 14.5739
October	15	89	74	48	34	890.5	15.1652	14.5738, 14.6449
November	17	74	55	54	39	450.7	13.5564	14.4925, 14.5143
December	15	96	61	44	38	909.7	15.2156	14.5503, 14.5984

**Table 9:** Accident Data in Jeddah.

Months	Saturday	Sunday	Monday	Tuesday	Wednesday	Thursday	Friday	$S_{N}^{2}$	$T_{k}{}_{N}$	NEWMA [λ = [0.08,0.12]]
January	426	601	596	586	574	583	407	7109.333	23.78843	[23.0023659,23.0365426]
February	487	812	525	476	421	498	413	18,339.81	26.44574	[23.2778358,23.4456463]
March	406	789	551	427	412	498	398	19,731.24	26.6508	[23.547673,23.8302647]
April	448	614	458	407	491	486	407	5203.619	22.91345	[23.4969351,23.720247]
May	423	611	518	457	427	482	412	4948.571	22.77254	[23.4389835,23.6065221]
June	530	590	563	475	479	511	372	5080.476	22.8463	[23.3915688,23.5152955]
July	493	623	511	587	587	528	396	5800.81	23.21808	[23.3776897,23.4796296]
August	453	652	579	578	552	503	427	6195.81	23.40279	[23.3796977,23.4704089]
September	491	546	503	498	488	517	410	1737.905	19.83877	[23.0964235,23.0346122]
October	378	412	422	413	382	456	373	880.8095	17.93394	[22.6834248,22.4225315]
November	394	533	449	380	393	405	394	2917.81	21.2914	[22.5720629,22.2867958]
December	402	576	517	397	388	419	307	7961.619	24.10591	[22.6947706,22.5050895]

## Data Availability

The data are given in the paper.

## Appendix A

#for laplace distribution need library (LaplacesDemon).
set.seed(5577)
rl<-c()
G<-c()
H<-c()
vt<-c()
var_SP<-c()
arl<-c()
n=7;Tk1=0.9165;mu.x=0.178;la=0.12;r0=370;a=-1.0827;b=2.8042;c=0.5678;Tk=0.00335
k1=2.787
k2=1.479
shift<-c(1.00,1.05,1.10,1.15,1.2,1.25,1.3,1.4,1.50,1.6,1.7,1.8,1.9,2.00,2.25,2.50,3.00,4)
for(l in 1:length(shift))
{
p1=shift[l]
for(j in 1:1000)
{
run=0
rep=0
for(i in 1:5000)
{
X=rnorm(n,0,1);X
sk=var(X);sk
x[i]=a+b*log(sk+c);x
var.x=var(x);var.x
ucl1=Tk+k1*sqrt((la/(2-la))*var.x);ucl1
lcl1=Tk-k1*sqrt((la/(2-la))*var.x);lcl1
ucl2=Tk+k2*sqrt((la/(2-la))*var.x);ucl2
lcl2=Tk-k2*sqrt((la/(2-la))*var.x);lcl2
#if (i==1){G[i]=la*SR[i])+(1-la)*mu.x}else{G[i])=la*SR[i]+(1-la)*G[i-1]}
if (i==1){H[i]=la*x[i]+(1-la)*mu.x} else{H[i]=la*x[i]+(1-la)*H[i-1]}
if(H[i]>ucl1|H[i]<lcl1){runs=i
break
}
if((H[i]>=lcl1 & H[i]<lcl2) | (H[i]>ucl2 & H[i]<=ucl1)){
rep=rep+1;
}
}
if(runs>0)
rl[j]=runs-rep
}
}
arl=mean(rl)
SDRL=sd(rl)
MDRL=median(rl)
print(cbind(arl,SDRL))
